# Ovule Gene Expression Analysis in Sexual and Aposporous Apomictic *Hypericum perforatum* L. (Hypericaceae) Accessions

**DOI:** 10.3389/fpls.2019.00654

**Published:** 2019-05-24

**Authors:** Giulio Galla, Andrea Basso, Simone Grisan, Michele Bellucci, Fulvio Pupilli, Gianni Barcaccia

**Affiliations:** ^1^Laboratory of Genetics and Genomics, Dipartimento di Agronomia, Animali, Alimenti, Risorse Naturali e Ambiente, University of Padova, Padua, Italy; ^2^Institute of Biosciences and Bioresources, Research Division of Perugia, National Research Council, Perugia, Italy

**Keywords:** *Hypericum perforatum* L., aposporous apomixis, LCM, RNAseq, ovule, RdDM

## Abstract

*Hypericum perforatum* L. (2*n* = 4*x* = 32) is an attractive model system for the study of aposporous apomixis. The earliest phenotypic features of aposporous apomixis in this species are the mitotic formation of unreduced embryo sacs from a somatic cell of the ovule nucellus and the avoidance of meiosis. In this research we addressed gene expression variation in sexual and apomictic plants, by focusing on the ovule nucellus, which is the cellular domain primarily involved into the differentiation of meiocyte precursors and aposporous embryo sacs, at a pre-meiotic developmental stage. Gene expression analyses performed by RNAseq identified 396 differentially expressed genes and 1834 transcripts displaying phenotype-specific expression. Furthermore, the sequencing and assembly of the genome from a diploid sexual accession allowed the annotation of a 50 kb sequence portion located upstream the HAPPY locus and to address the extent to which single transcripts were assembled in multiple variants and their co-expression levels. About one third of identified DEGs and phenotype-specific transcripts were associated to transcript variants with alternative expression patterns. Additionally, considering DEGs and phenotype-specific transcript, the co-expression level was estimated in about two transcripts per locus. Our gene expression study shows massive differences in the expression of several genes encoding for transposable elements. Transcriptional differences in the ovule nucellus and pistil terminal developmental stages were also found for subset of genes encoding for potentially interacting proteins involved in pre-mRNA splicing. Furthermore, the sexual and aposporous ovule transcriptomes were characterized by differential expression in genes operating in RNA silencing, RNA-mediated DNA methylation (RdDM) and histone and chromatin modifications. These findings are consistent with a role of these processes in regulating cell fate determination in the ovule, as indicated by forward genetic studies in sexual model species. The association between aposporous apomixis, pre-mRNA splicing and DNA methylation mediated by sRNAs, which is supported by expression data and by the enrichment in GO terms related to these processes, is consistent with the massive differential expression of multiple transposon-related sequences observed in ovules collected from both sexual and aposporous apomictic accessions. Overall, our data suggest that phenotypic expression of aposporous apomixis is concomitant with the modulation of key genes involved in the two interconnected processes: RNA splicing and RNA-directed DNA methylation.

## Introduction

Apomixis defines a subset of plant reproductive strategies which, unlike sexual reproduction, allow the inheritance of the maternal genome through seeds, without genetic recombination, and syngamy (Nogler, 1984). This asexual mode of seed formation has enormous economic and social potential in agriculture, as genetically fixing highly heterozygous genotypes through apomixis would have tremendous advantages for those crops which are commercialized as hybrid F1 seed (Vielle-Calzada et al., 1996a). Although apomixis is relatively common in nature, this trait has never been reported for the major crop species (Carman, 1997).

Known apomictic developmental pathways are traditionally classified into two main categories: sporophytic and gametophytic (Koltunow and Grossniklaus, 2003). In sporophytic apomicts, somatic cells from the ovule nucellus differentiate to generate multiple embryos, which coexist with the sexually-formed sibling and share its endosperm. Conversely, plants reproducing via gametophytic apomixis (Nogler, 1984) possess the ability to differentiate viable unreduced embryo sacs from unreduced cells of the ovule (i.e., unreduced meiocytes, somatic cells).

Both hybridization and polyploidy have wide-ranging effects on the chromosomal (Sanchez-Moran et al., 2001; Kantama et al., 2007), genomic (Soltis and Soltis, 1999; Pikaard, 2001), and transcriptomic (Adams et al., 2004; Adams, 2007) levels and as such have been suggested as possible mechanisms with the ability to elicit apomictic reproduction (Carman, 1997; Grossniklaus et al., 2001). Accordingly, heterochronic expression of genes involved in sexual reproduction has been associated to the apomictic developmental pathway by several authors (Grimanelli et al., 2003; Sharbel et al., 2010).

The key biological features of apomixis, with particular reference to aposporous type of gametophytic apomixis, are: (i) embryo sac development from a somatic cell of the ovule nucellus, (ii) parthenogenesis (i.e., fertilization-free embryo development); and (iii) the formation of viable endosperm either via fertilization-independent means or following fertilization with a sperm cell (Koltunow and Grossniklaus, 2003). These features deviate from sexuality as the capability to develop an embryo sac is strictly restricted to the reduced functional megaspore (FM) deriving from meiosis, and failure of the meiotic program in obligate sexual species is not accompanied by the initiation of embryo sac development from cell lineages of the ovule other than the FM. The ovule of higher plants is a multicellular structure composed by at least four cellular domains morphologically distinguishable from the proximal–distal axis: the funiculus, which attaches the ovule to the placenta; the chalaza, which forms the integuments; the nucellus, from which the progenitors of the MMC are specified; the integuments, which surround the nucellus. The cellular domain from which female gametes are generated is composed by a relatively small group of cells embedded within the ovule nucellus. While MMC founder cells are typically specified from the two innermost layers of nucellus, a single FM is non-randomly specified among the four meiocytes deriving from the MMC through meiosis. The progenitors of unreduced embryo-sacs (AI) found in ovules of aposporous apomictic plants also differentiate from the nucellus (Mártonfi et al., 1996; Galla et al., 2011).

Despite its prime importance for plant reproduction, the molecular mechanisms that control the somatic-to-reproductive transition are still poorly understood. Among biological macro-processes potentially involved in this process, the epigenetic regulation of gene expression and hormonal homeostasis seems to play fundamental roles. Epigenetic regulation of gene expression either by DNA methylation and post-transcriptional gene silencing (PTGS) is required for proper ovule development (Garcia-Aguilar et al., 2010). Specification of gametophyte precursors in *Arabidopsis thaliana* is controlled by the expression of AGO9, which restricts the specification of gametophyte precursors in a dosage-dependent, non-cell-autonomous manner (Olmedo-Monfil et al., 2010; Armenta-Medina et al., 2011; Singh et al., 2011; Tucker et al., 2012). DNA methylation mediated by small RNAs (RdDM) is essential for gametophyte development and loss-of-function mutants for genes involved in this pathway produce aberrant cell fate establishment within the ovule, with phenotypes that are strikingly reminiscent of apomictic development (Hernandez-Lagana et al., 2016). These findings suggest that expression of genes involved in the establishment sexual-to-aposporous dichotomy are epigenetically regulated.

*Hypericum perforatum* L., an invasive perennial herb widely distributed in a variety of habitats, is regarded as a serious weed in many countries (Robson, 2002; Nürk et al., 2013). The mode of reproduction in *H. perforatum* is highly dynamic, and biotypes span from almost complete sexuality to nearly obligate apomixis (Noack, 1939; Davis, 1967; Mártonfi et al., 1996; Matzk et al., 2001; Barcaccia et al., 2007; Galla et al., 2011, 2015). From an evolutive point of view, *H. perforatum* most likely originated by autopolyploidization or secondary hybridization and introgression between distinct gene pools within *H. perforatum* and with the sister species *H. maculatum* (Scheriau et al., 2017). Apospory in *H. perforatum* is inherited as a dominant simplex locus (HAPPY), which segregates from the genetic factors controlling parthenogenesis (Schallau et al., 2010). This plant species is regarded as an interesting model for apomixis research due to a number of interesting traits, including: (i) the versatile and dynamic mode of reproduction; (ii) the relatively small genome size (0.325 pg/1Cx); (iii) the short generation time (i.e., a flowering stage/year in wild type population and up to three flowering stage/year in greenhouse); (iv) the availability of a large number of morphologically distinct ecotypes; (v) the self-compatibility and high seed set (Matzk et al., 2001; Barcaccia et al., 2007).

Since the earliest study of Vielle-Chalzada in sexual and apomictic ovaries of *Pennisetum* (Vielle-Calzada et al., 1996b), comparative transcriptomics have been carried out in several aposporous apomictic systems including *Poa pratensis* (Albertini et al., 2004), *Pennisetum ciliare* (Singh et al., 2007), *Panicum maximum* (Yamada-Akiyama et al., 2009), *Paspalum simplex* (Polegri et al., 2010), *Hieracium praealtum* (Okada et al., 2013), *Ranunculus auricomus* (Pellino et al., 2013), and *Boechera gunnisoniana* (Schmidt et al., 2014). Although a few comparative transcriptomic studies have been already performed in *H. perforatum* (Galla et al., 2013, 2015, 2017), these investigations were carried out by focusing at the organ level (e.g., at pistil level), implying a relatively low resolution in detecting transcriptional differences occurring within the ovule nucellus. Also, as previous studies focused on flower developmental stages spanning female sporogenesis and gametogenesis (Galla et al., 2017), the transcriptional changes occurring in the ovule nucellus before the failure of meiosis and differentiation of aposporous initials in *H. perforatum* are largely unexplored. To gain additional insight on these transcriptomic changes and define the biological processes potentially leading to the aposporous developmental program, we performed an RNAseq gene expression analysis on the ovule nucellus collected from sexual and aposporous plant accessions by Laser Capture Microdissection (LCM).

## Materials and Methods

### Plant Materials

Whole genome sequencing efforts were performed by using an *H. perforatum* L. diploid (2*n* = 2*x* = 16) sexual accession (ACC ID:OBUPD-D1) kindly provided by the Padova Botanical Garden^1^ (Supplementary Table S1). Naturally occurring *H. perforatum* tetraploids (*2n* = 4*x* = 32) were selected from two local populations (Acc ID: HP1 and HP4) collected in Northern Italy, province of Belluno (Barcaccia et al., 2006). *H. perforatum* L. induced tetraploids (2*n* = 4*x* = 32) were kindly donated by Dr. T. F. Sharbel (IPK-Gatersleben). Induced tetraploids were generated by colchicine application as described by Schallau et al. (2010). The reproductive mode of all *H. perforatum* accessions was estimated by flow cytometric screening of 48 single seeds as described by Matzk et al. (2001) and Schallau et al. (2010). The phenotype of investigated plant accessions was defined as sexual, when all investigated seeds displayed 4:6 embryo:endosperm C-values. For naturally occurring *H. perforatum* tetraploid accessions only plant individuals with >96% apomixis were considered. For the purposes of this research, BIII hybrids characterized by 6:8 or 6:10 embryo:endosperm C-values were classified as apomictic. The frequency of BIII hybrids ranged from 0 to 44%, with a median value of 14%. Sexual plants adopted for the RNAseq analysis were selected among induced tetraploids, whereas apomicts were selected from both naturally and induced tetraploid accessions (Supplementary Table S1). RNAseq analyses were performed by using nine sexual and nine apomictic accessions (Supplementary Table S1).

### Genome Sequencing and Assembly

Genomic DNA was extracted using the CTAB protocol (Doyle and Doyle, 1990) and quantified with the Qubit dsDNA BR Assay kit (Life Technologies). DNA purity and integrity were assessed at the Nanodrop 1000 spectrophotometer (Thermo Scientific) and by capillary electrophoresis on a 2200 TapeStation (Agilent Technologies), respectively. For genome assembly, the presence of high molecular weight DNA was verified using Field-inversion gel electrophoresis (FIGE) and subsequently DNA fragments >40 kb were enriched using BluePippin (Sage Science).

The Chromium Gel Bead and Library Kit (10X Genomics) and the Chromium instrument (10X Genomics) were used to prepare linked-reads WGS libraries from 0.625 ng of high molecular weight DNA of the sexual/diploid sample. The generated barcoded library was sequenced on an Illumina HiSeqX Ten using 151 nt reads in paired-end for a total of 41.7 Gbp (138170412 fragments). Genomic linked reads were assembled using the Supernova assembler version 2.1.0 (Weisenfeld et al., 2017) using default parameter excepted for –max-reads that was set to 170000000. Final scaffolds were produced using Supernova mkoutput pseudohap option discarding scaffolds shorter than 300 bp. The annotation of genomic contigs matching the HAPPY locus has been performed by BLASTN, by using the gene sequences annotated in contig HM061166 to query the genome assembly. The newly identified genomic contigs were annotated with BLASTX and by using the nr database^2^.

### Tissue Embedding, Laser Microdissection, RNA Extraction, and Amplification

Flower buds of approximately 3.0 mm, corresponding to *Arabidopsis* flower stage 11 (Galla et al., 2011) were collected, immediately placed on a glass Petri dish containing ice-cold acetone and opened to pick up the ovary under a dissecting microscope. Ovaries were stored over night at 4°C in fresh acetone. After vacuum infiltration for 30–60 min, acetone was replaced with consecutive washings for 20 min in ice using 3:1, 1:1, and 1:3 mixtures of acetone:xylene, followed by two changes of pure xylene for 20 min each. Then, paraffin infiltration was performed by moving ovaries to 60°C and washing them at 20-min intervals in 1:3, 1:1, and 3:1 mixtures of paraffin:xylene. Samples were washed other two times for 1 h each in pure paraffin at 60°C, lodged in square mesh cassettes, solidified at room temperature and stored at 4°C.

Ovaries were cut into 10-μm sections with a Leica Jung Autocut 2055 microtome, placed on Zeiss MembraneSlide 1.0 PEN, and floated on diethylpyrocarbonate (DEPC) treated water at 40°C on a slide warming tray until the sections stretched. Sections were deparaffinized in three changes of xylene, dried at RT for 10 min and immediately dissected with a Zeiss PALM Microbeam IV equipped with a Axio Observer inverted microscope. After that 250.000 μm^2^ of tissue sections were collected in a single tube, 20 μl of lysis buffer from the Stratagene Absolutely RNA^®^ Nanoprep Kit (Agilent) were added, and RNA was isolated according to the manufacturer’s instructions. RNA integrity and concentration were assayed on a 2100 Bioanalyzer with the RNA 6000 Pico Kit. cDNA synthesis and amplification were performed by pooling the RNAs from nine sexual and nine apomictic accessions in three sexual and three apomictic samples. cDNA synthesis and amplification were performed by using the AMBION MessageAmp^TM^ II aRNA Amplification Kit. Libraries. The TruSeq Stranded Total RNA Sample Prep Kit was used to prepare the libraries from 50 ng of aRNA. The generated barcoded library was sequenced on an Illumina NextSeq500 using 151 nt reads in paired-end.

### Transcriptome Assembly, Annotation, and Gene Expression Analysis

*De novo* assembly of transcript sequences was done with the following pipeline. All reads with more than 10% of undetermined bases (Ns) or with more than 50 bases called with a phred-scored quality lower than 7 (probability of wrong call > 20%) were discarded. Following the reads quality filtering, sequence adapters were clipped by using scythe^3^. 3′ ends of reads were quality trimmed with a quality threshold of 20 over a window of 10 bases with sickle^4^. Reads shorter than 20 bp were discarded. Read Coverage of transcripts in libraries were digitally normalized using a k-mer abundance approach with insilico_read_normalization.pl script of Trinity software setting a k-mer = 20 and a max depth of 60. Assembly of the transcripts was performed using Velvet/Oases pipeline in multi k-mer mode, using k-mers from 19 to 95 with in 4 bp steps and merging the assembled transcripts. Finally, merged transcriptome assemblies were clustered using EvidentialGene software to obtain a high-quality reference transcriptome assembly, removing potential artifacts and clustering redundant transcripts.

The annotation of assembled sequences was performed as described in Galla et al. (2015, 2017). Briefly, to annotate all assembled unigenes, a BLASTX-based approach was used to compare the *Hypericum* sequences to the nr database downloaded from the NCBI^2^. The GI identifiers of the best BLASTX hits, with E-values ≤ 1 E-09 and similarities ≥ 70%, were mapped to the UniProtKB protein database^5^ to extract Gene Ontology (GO^6^) terms for further functional annotations. BLAST2GO software v1.3.3^7^ (Conesa et al., 2005) was used to reduce the data to the GOslim level^8^ and perform basic statistics on ontological annotations as previously reported (Galla et al., 2009). Annotations for genes involved in plant reproduction were retrieved from Galla et al. (2017). Annotations for genes involved in hormonal homeostasis were retrieved from AmiGO 2^9^.

For Gene Expression analysis, mapping and sequence counts were performed with the software CLC Genomics Workbench V 7 (Qiagen), with default parameters and by using the *de novo* transcriptome as reference. Differentially expressed genes (DEGs) were identified by using the software empirical analysis of DGE (Robinson and Oshlack, 2010) implemented in the CLC Genomics Workbench V 7 (Qiagen), and by adopting the FDR (*p*-value ≤ 0.05) and Bonferroni *p*-value correction (*p*-value ≤ 0.05). Sexual samples were adopted as reference. Principal component analysis and heat maps were generated with the software T-mev^10^. PCoA were generated by using all transcripts with expression ≥ 20^th^ percentile and DEGs (Bonferroni *p*-value ≤ 0.05), respectively. Heat maps were generated with the HCL algorithm, by using Manhattan distances and average linkage clustering.

### Expression Analysis by Real-Time qPCRs and *in situ* Hybridization Assays

Plant materials were selected according to the genetic and cyto-histological bases of apospory recently described for *H. perforatum* (Schallau et al., 2010; Galla et al., 2011). Pistils were collected separately from a minimum of five plant accessions (Supplementary Table S1). Total RNA was extracted from collected pistils using the Spectrum^TM^ Plant Total RNA Kit (Sigma-Aldrich), by following the protocol provided by the manufacturer. The eventual contamination of genomic DNA was avoided by using optional DNase I (Sigma-Aldrich) treatment. The abundance and pureness of RNAs were assessed using a NanoDrop 2000c UV-Vis spectrophotometer (Thermo Scientific, Pittsburgh, PA, United States). cDNA synthesis was performed using the RevertAid First Strand cDNA Synthesis Kit (Thermo Scientific), following the instructions of the supplier. Primers used in the Real-Time RT-PCR experiments are reported in Supplementary Table S12. Expression analyses were performed using StepOne thermal cyclers (Applied Biosystems), equipped with 96-well plate systems, respectively, with SYBR green PCR Master Mix reagent (Applied Biosystems). The amplification efficiency was calculated from raw data using OneStep Analysis software (Life Technologies). Amplification performance expressed as fold change was calculated with the ΔΔCt method using *HpTIP4* as a housekeeping gene (Pfaffl, 2001). Error bars indicate the standard error observed among the five biological replicates. *In situ* hybridization assays were performed as described by Brewer et al. (2006). For the synthesis of probes, approximately 50 ng of cDNA from apomictic pistils was amplified using specific primer pairs (Supplementary Table S12). Amplicons were purified with the QIAquick PCR Purification Kit (QIAGEN) and sequenced on an ABI3100 automated sequencer to confirm the target specificity. Purified amplicons were then diluted 1:20 and amplified with T7- and T3-tailed primers (in separate reactions) to incorporate the T7 and T3 promoter (Supplementary Table S12). Both probes were labeled using a Roche DIG RNA labeling kit. Detection was performed following the Roche DIG detection kit instructions using anti- DIG AP and NBT/BCIP as substrates. Images were acquired with a Leica DM4000B digital microscope, equipped with a Leica DC300F camera and Leica Image Manager 50 software (Leica Microsystems).

### CpG Methylation Analysis

Methylation analysis of TEs-related sequences was performed by using the OneStep qMethyl Lite Kit (Zymo Research). Genomic DNA was extracted from single pistils collected from three sexual and three aposporous apomictic plant accessions. DNA extraction were performed by using the QIAamp DNA Investigator Kit (Qiagen), by following guidelines provided for the isolation of total DNA from tissues. The abundance and pureness of DNA samples were assessed using a NanoDrop 2000c UV-Vis spectrophotometer (Thermo Scientific, Pittsburgh, PA, United States).

### Data Availability

Raw sequences files were made available for download from SRA with the following Accession Nos. SAMN10880815, SAMN10880814, SAMN10880813, SAMN10880812, SAMN10880811, SAMN10880810. The Transcriptome Shotgun Assembly project has been deposited at DDBJ/EMBL/GenBank under the Accession No. GHFN00000000. The version described in this paper is the first version, GHFN01000000. The *H. perforatum* genome was submitted as WGS submission with the Accession No. SOPF00000000. The expression data discussed in this publication have been deposited in NCBI’s Gene Expression Omnibus (Edgar et al., 2002) and are accessible through GEO Series Accession No. GSE128923^11^.

## Results

### Assembly and Annotation of the *H. perforatum* Ovule Transcriptome

**FIGURE 1:**
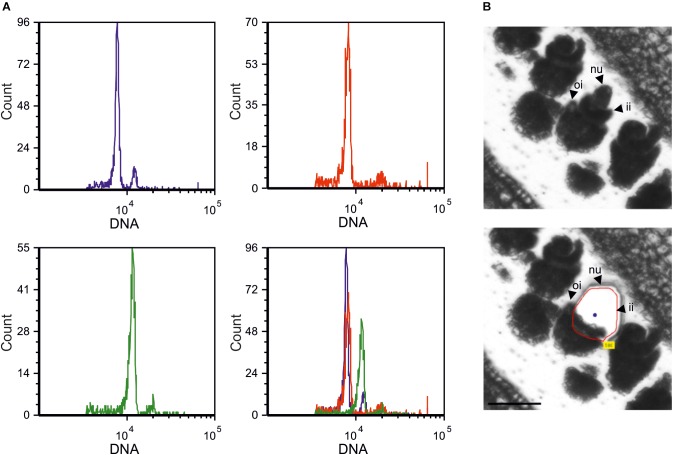
*Hypericum perforatum* main reproductive strategies and tissue type investigated in this study. **(A)** DNA histograms displaying estimates of DNA content by using *H. perforatum* single seeds, as assessed by FCSS. Blue: seed originated by sexual reproduction (4:6 embryo:endosperm C-values); red: single seed originated by autonomous apomixis (4:8 embryo:endosperm C-values); green: BIII hybrid (6:8 embryo:endosperm C-values). The overlay of these three profiles is also shown. **(B)** Ovule developmental stage adopted for Laser-Capture Microdissections (upper panel). The remaining tissue is displayed in the bottom panel. Dissections were performed by targeting the ovule nucellus (red line). nu: nucellus; ii: internal integuments; oi: outer integuments. Scale bar: 50 mm.

Laser Capture Microdissections (LCM) were performed on ovules at a pre-meiotic developmental stage (Figure 1), collected from sexual and aposporous apomictic *H. perforatum* accessions (Supplementary Table S1). Microdissections were performed by focusing on the nucellus on ovules in which integuments were already differentiated, but not completely overlapping the nucellus (Figure 1B). A summary of RNAseq data is reported on Supplementary Table S2. The sequencing reaction originated, on average, 177.7 million reads per library (88.8 pair-ends). The assembly of all high-quality reads in a single reference transcriptome originated 67022 sequence contigs. The length of the assembled sequences ranged from 201 to 6743 bp. The average and median sequence length were 691 and 590 bp, respectively. The N50 of the transcriptome was estimated in approximately 800 bp (Table 1).

**Table 1:** Summary statistics of the sequence assembly and functional annotation for *Hypericum perforatum* ovule transcriptome.

Transcriptome assembly	
Total sequences	67022
Min sequence length	201
Max sequence length	6743
Average sequence length	691,5
Median sequence length	590
N50 length	814
**Functional annotation**	
Transcripts with BLAST matches	51666 (77.09%)
Arabidopsis gene models	15795
Transcripts without BLAST matches	15356 (22.91%)
UNIPROTKBID	51555 (76.92%)
Gene ontology	46846 (69.90%)


*BLAST matches are referred to the *Arabidopsis thaliana* proteome TAIR10.*

The polyploid nature (2*n* = 4*x*) of investigated *H. perforatum* accessions, together with the large size of our transcriptome assembly, prompted us to investigate the extent to which single transcripts were assembled in multiple variants. To address this issue, we sequenced the genome of a sexual unrelated *H. perforatum* (2*n* = 2*x* = 16; 0.325 pg/1Cx) accession and used the assembled draft for the alignment of all DEGs and phenotype-specific transcripts (Table 2 and Supplementary Table S3). In its current version, the draft sequence of the *H. perforatum* genome is about 350 Mb in size, with a scaffold N50 of 63 kb. The number of assembled scaffolds is close to 37,000, with an average length of about 10 kb (Table 2). A preliminary investigation concerning the completeness of the assembly was performed by using the benchmarking universal single-copy orthologs (BUSCO) analysis. Using the embryophyta_odb9 as reference database, we estimated an 85.7% completeness, 12.8% of duplicated (N: 184/1440) and about 5% (75/1440) of fragmented BUSCOs. 9.1% of BUSCOs were missing from the assembled genome (N:131 out 1440). By taking advantage of the *H. perforatum* genome draft, we could efficiently discriminate transcript variants (e.g., potential alleles) by co-aligning to the same genomic locus, from gene products related to orthologous or paralogous gene loci (see below).

**Table 2:** Summary of sequencing and assembly data for the *H. perforatum* (2*n* = 2*x*) genome draft.

Summary of sequencing data	R1	R2
Number of reads	138,170,412	138,170,412
Total Bp	20,863,732,212	20,863,732,212
Q20	97.69	92.52
Q30	94.28	85.67
GC%	40.71	39.37
Expected Coverage	130X	
**Summary of assembly data (scaffolds; length > 300 bp)**		
Assembly size (bp)	354,935,916	
# Scaffolds	36,713	
Scaffolds N50 (bp)	63,135	
Average scaffolds length (bp)	9,667	
Largest scaffolds length (bp)	985,864	
# Gaps	5,613	
Gap size (bp)	2,862,790	


*The summary of sequencing data refers to the R1 and R2 reads.*

The annotation of the ovule transcriptome was performed by using the *Arabidopsis thaliana* proteome (TAIR10) as reference. As shown in Table 1, we annotated 51666 transcripts (77% of the assembled transcripts), matching 15795 Arabidopsis gene models (e-value cut-off: 1.0E-9). 46846 transcripts (about 70% of the transcriptome) were assigned with one or more GO ontological term (Table 1). According to the GO-slim nomenclature (Supplementary Figure S1), a large portion of the *H. perforatum* ovule transcriptome is depicted to metabolic and cellular process (GO:0008152 and GO:0009987, respectively). Cellular component organization or biogenesis (GO:0008150), developmental process (GO:0032502), and response to stimulus (GO:0050896) were also highly represented in our datasets. At this ontological level (GO-slim, level2), the GO term reproduction (GO:0000003) was associated to about 5500 transcripts, accounting for approximately 12% of the ovule transcriptome (Supplementary Figure S1).

### Gene Expression Analysis Reveals an Enrichment of Processes Related to RNA-Dependent DNA Biosynthesis and RNA Processing in Ovules Collected From Aposporous Accessions

**FIGURE 2:**
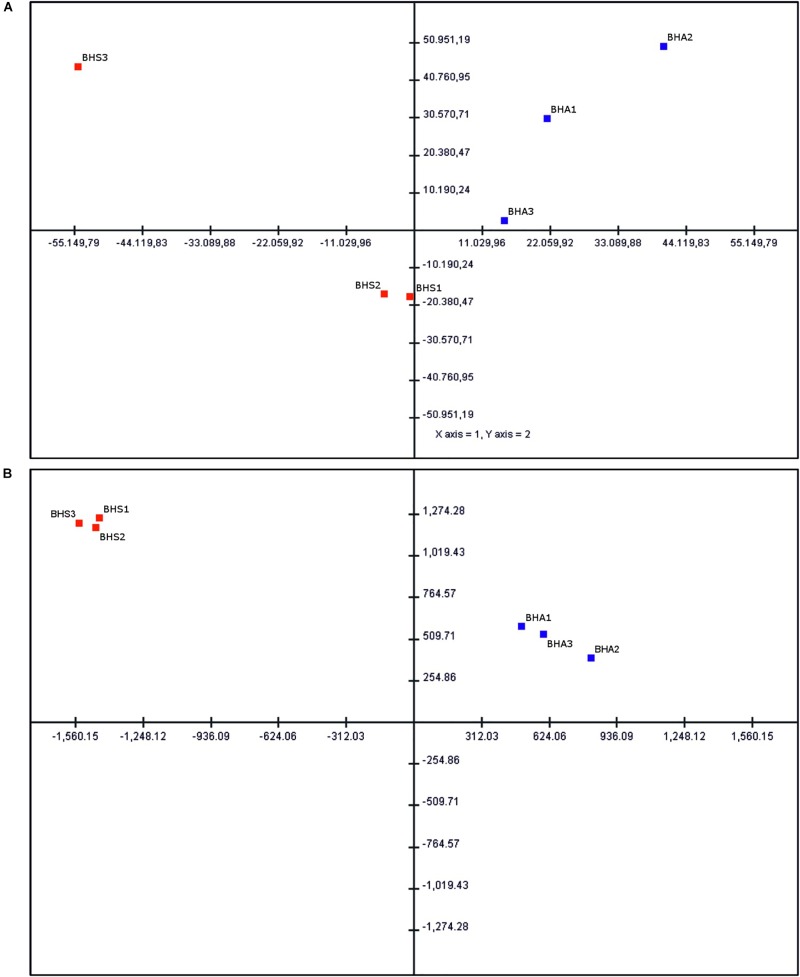
Principal component analysis (PCA) of ovule-expressed transcripts. **(A)** Statistical analysis performed by using all expressed transcripts. The percentage variation explained by the two axes is about 44%. **(B)** Statistical analysis performed by using DEGs and phenotype-specific transcripts. The percentage variation explained by the two axes is about 96%. Sexual samples (BHS1-3) are shown as red squares, while aposporous apomictic samples (BHA1-3) are shown as blue squares.

Gene expression (GE) analyses were performed by using the ovule transcriptome assembled *de novo* as reference. The mapping of high-quality reads to this reference aligned, on average, 50% of sequence reads (Supplementary Table S2). However, the average number of mapped reads was about 88 million single reads per sample. The percentage of mapped reads varied from 45 to 55%, with no obvious correlation with the reproductive behavior of considered accessions. A principal component analysis (PCA) computed by using all expression data points separated sexual samples from the apomictic ones, with the two first principal components explaining approximately 43.7 of the overall variance (Figure 2).

As shown in Table 3, 396 transcripts (Bonferroni *p*-value ≤ 0.05) were found to be differentially expressed between the two phenotypes. More in detail, and by using the sexual samples as reference, 312 (FDR: 1129) transcripts were found up-regulated whereas 85 (FDR: 437) were found down-regulated (Table 3 and Supplementary Table S3). Remarkably, 119 transcripts were exclusively present in sexual (Bonferroni *p*-value ≤ 0.05; N: 25) or aposporous samples (Bonferroni *p*-value ≤ 0.05; N: 94). By considering the FDR *p*-value correction or no *p*-value correction, our estimates of phenotype-specific transcripts (e.g., the sum of APO-specific and SEX-specific transcripts) raised to 376 and 1456, respectively (Table 3 and Supplementary Table S4).

**Table 3:** Differentially expressed genes.

DEGs	Expression	No.
	profile	
396 (1512)	Up	312 (1075)
	Down	85 (437)
119 (376/1456)	APO	94 (300/1138) (1075)
	SEX	25 (76/318)


*For each comparison the table reports on the number of identified differentially expressed unigenes in the aposporous samples (test) with respect to the sexual samples (reference). Three biological replicates were adopted for both test (t) and reference (r) samples. For each comparison the number of up- and down-regulated unigenes is also indicated for each class of DEGs. Numbers in brackets indicate the number of DEGs resulting by the adoption of the FDR *p*-value correction.*

The *in silico* mapping of genes located in the HAPPY (Hypericum APOSPORY) locus associated to apospory in *H. perforatum* (NCBI Accession No. HM061166) identified nine contigs in the draft sequence of the *H. perforatum* genome (Supplementary Table S5). While seven contigs were shorter than 10 kb in length, the two contigs: 62189 and 62786 accounted for 58,364 and 89,182 bp, respectively. Sequence identity between gene regions predicted from HM061166 and the corresponding genome sequences ranged from 93 to 100%, with an average value of 98%. The lowest sequence identity was scored by *ARI-T* gene locus (Supplementary Table S5). The largest contig (62786) corresponded to sequence portion of HM061166 bracketed by the two genes *PAT1* and *RINGH2*, which includes the locus homologous to the *A. thaliana* gene *ARI7*. Both gene composition and gene order were perfectly conserved between contig 62786 and the corresponding sequence portion of HM061166. The only exception to this was the miss annotation of the transposon related gene *RT3* in contig 62786. Noteworthy, the gene sequence corresponding to the first locus predicted from HM061166 (e.g., *HK1*) aligned with a terminal portion of contig 62189, ranging from positions 52192 to 58131. The annotation of contig 62189 predicted nine genes with no homology relationship with genes annotated in the BAC clone HM061166, except for *HK1*. Hence, we considered likely that the 50 kb sequence portion upstream the gene locus *HK1* in contig 62189 represents the genomic sequence upstream of the HAPPY locus in the *H. perforatum* genome (Supplementary Table S5). Based on the alignment of transcript variants to the 9 genomic contigs, we could verify that most predicted genes in this region (N: 24/33) are expressed in pre-meiotic ovule nucellus. However, the transcript Locus_2_Transcript_538734_835163_Confidence_0.000_ Length_588, encoding for a light harvesting chlorophyll a/b-binding protein (Lhcb2), was the only DEG associated to HAPPY locus (FDR: 4.2E-3). The *ARIADNE* locus predicted from the contig 62786 (contig region 20985–22397 bp) matched four transcript variants (Supplementary Table S5) displaying no expression differences in our datasets. No transcripts were associated to the annotated region corresponding to the *ARI-T* locus (contig region 23718–24280 bp).

**FIGURE 3:**
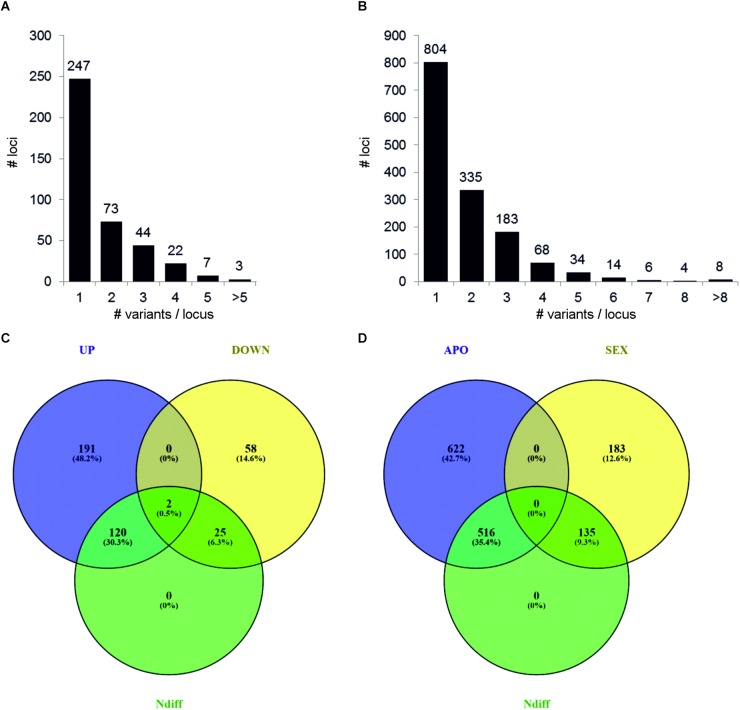
Distribution of single and multiple transcript variants per locus. Metrics are referred to genomic loci containing at least one DEG **(A)** or phenotype-specific transcript **(B)**. Transcript variants were defined as transcripts aligning into the same genomic locus. DEGs were defined by considering the Bonferroni *p*-value correction (*p* ≤ 0.05). Phenotype-specific transcripts were defined as transcripts expressed in the three samples of a given phenotype and absent in the three samples of the alternative phenotype. The Venn diagrams show the number of loci associated to transcripts with alternative expression patterns. Loci associated to up-regulated and down-regulated DEGs are shown in **(C)**, while APO-specific and SEX-specific transcripts are shown in **(D)**.

In a different approach, the *H. perforatum* genome sequence was exploited to efficiently discriminate transcript variants (e.g., potential alleles) co-mapping into the same genomic locus, from gene products related to orthologous or paralogous gene loci. Detailed annotations for all identified DEGs (Bonferroni *p*-value ≤ 0.05) and phenotype-specific transcripts are reported on Supplementary Tables S3, S4. Data are referred to genomic loci containing at least one modulated transcript. We therefore focused our statistics on co-expression of multiple transcript variants to DEGs and transcripts exclusively found in aposporous or sexual samples. In the former case, the average number of multiple transcript variants was equal to 1.69 (range: 1–8), while we estimated an average number of 1.86 transcripts/locus (range: 1–13) for phenotype-specific transcripts. In both cases, about two thirds of transcriptionally modulated transcripts were present as single variants (Figure 3A,B). More in detail, the number of loci associated to transcripts with multiple expression patterns (N ≥ 2) was equal to 120 (38.9%) and 25 (29.5%) for up- and down-regulated DEGs (Figure 3C). For APO- and SEX-specific transcripts, the number of loci associated to multiple expression patterns was equal to 516 (45.3%) and 135 (42.4), respectively (Figure 3D).

The analysis of GO enriched terms provided an overview of biological processes and molecular functions that characterize the transcriptional changes seen in ovules (Table 4 and Supplementary Table S6). GO terms were filtered to the most specific ones to permit a clear, non-redundant, picture of over- and under-represented processes and functions. Among up-regulated transcripts, we found a significant enrichment for the GO term: RNA-dependent DNA biosynthetic process (FDR *p*-value: 8.4E+08). This ontological term was found to be enriched in both up-regulated and in down-regulated transcript sets (Bonferroni and FDR), as well as among APO-specific transcripts (FDR *p*-vale: 1,2E-02). Other enriched terms found in upregulated transcripts (FDR *p*-value ≤ 0.05) were RNA processing, glyceraldehyde-3-phosphate metabolic process, organelle organization and multicellular organism development (Table 4 and Supplementary Table S6). Among down-regulated transcripts, enrichments were found for the GO terms: synaptonemal complex assembly, plant-type cell wall modification and gene expression. Noticeably, while no enrichment was found in SEX-specific transcripts, the APO-specific ones were depleted in multiple terms, including pollen tube growth, cell cycle process, vegetative to reproductive phase transition of meristem and regulation of gene expression, epigenetic, among others (Supplementary Table S6).

**Table 4:** Enrichment analysis of GO terms over or under represented in *H. perforatum* ovule DEGs.

GE pattern (*p*-value correction)	Vocabulary	GO term	Description	Enrichment	FDR	*p*-Value	#Annot test	#Annot ref	#notAnnot test	#notAnnot ref
UP (Bonferroni *p*-value ≤ 0.05)	BP	GO:0006278	RNA-dependent DNA biosynthetic process	OVER	8.4E+08	2.3E+06	11	200	209	50909
	
	MF	GO:0003964	RNA-directed DNA polymerase activity	OVER	8.4E+08	1.7E+07	11	194	209	50915
	
DOWN (Bonferroni *p*-value ≤ 0.05)	BP	GO:0007130	Synaptonemal complex assembly	OVER	1.8E-02	5.0E+09	2	1	65	51261

UP (FDR *p*-value ≤ 0.05)	BP	GO:0006278	RNA-dependent DNA biosynthetic process	OVER	4.9E+05	2.7E+02	24	187	843	50275
		GO:0006396	RNA processing	UNDER	7.5E+11	1.8E+10	13	2271	854	48191
		GO:0019682	Glyceraldehyde-3-phosphate metabolic process	UNDER	2.7E-03	8.4E+06	1	885	866	49577
		GO:0006996	Organelle organization	UNDER	3.8E-03	1.4E+11	67	6266	800	44196
		GO:0007275	Multicellular organism development	UNDER	1.0E-02	4.2E+10	105	8708	762	41754
		GO:1901566	Organonitrogen compound biosynthetic process	UNDER	1.2E-02	5.1E+11	68	6143	799	44319
		GO:0043604	Amide biosynthetic process	UNDER	3.3E-02	1.8E+12	11	1697	856	48765
		GO:0009117	Nucleotide metabolic process	UNDER	3.5E-02	2.0E+11	21	2542	846	47920
		GO:0040029	Regulation of gene expression, epigenetic	UNDER	4.7E-02	2.9E+11	7	1299	860	49163
	
	MF	GO:0003964	RNA-directed DNA polymerase activity	OVER	3.4E+06	1.4E+03	24	181	843	50281

DOWN (FDR *p*-value ≤ 0.05)	BP	GO:0007130	Synaptonemal complex assembly	OVER	1,1E-03	3,20E+08	3	0	349	50977
		GO:0006278	RNA-dependent DNA biosynthetic process	OVER	1,0E-02	1,73E+11	9	202	343	50775
		GO:0009827	Plant-type cell wall modification	OVER	3,1E-02	8,18E+10	12	441	340	50536
		GO:0010467	Gene expression	UNDER	4,1E-02	1,16E+12	30	7962	322	43015
	
	MF	GO:0003964	RNA-directed DNA polymerase activity	OVER	9,8E-03	1,38E+11	9	196	343	50781


*Only overrepresented and underrepresented GO terms are indicated. Enrichment analyses were performed by using each set of DEGs (GE pattern) as test sample and the whole transcriptome as reference. For each set of DEGs and GO vocabulary (Ontology) the table reports on the enriched GO term, the GO description (Description), the nature of enrichment (enrichment), the *p*-value associated to the statistical test and the FDR corrected *p*-value. For each GO term, the number of annotated (#Annot) and non-annotated (#nonAnnot) transcripts in the test (Test) and reference datasets (Refs) is also indicated.*

Interestingly, the only over-represented GO term in down-regulated DEGs (FDR *p*-value: 1.8E-02) was synaptonemal complex assembly (GO:0007130). As apomixis process is expected to be associated with transcriptional changes on reproductive-related genes, we annotated our transcriptome according to the following major classes: cell specification, sporogenesis, gametogenesis, and embryogenesis and selected all transcripts displaying transcriptional changes in the ovule nucellus datasets (Supplementary Table S7). More in details, we found five transcript variants potentially associated to cell fate specification, 11 transcript variants potentially involved in sporogenesis and 30 transcripts associated to later developmental processes, including gametogenesis (n:12) and embryo development (n:18). The transcript variants associated to the cell fate specification were up-regulated in the aposporous dataset or found exclusively expressed in nucellus of aposporous plant accessions. Among these, we found the orthologs of Arabidopsis *BEL1*-like homeodomain 1 and *MYB* domain protein 66 (Supplementary Table S7). Similarly, the datasets represented by transcripts potentially involved in gametogenesis and embryo development displayed a clear disproportion between transcripts displaying higher expression in aposporous-derived ovules (including Up-regulated DEGs and APO-specific) and transcripts with the opposite expression pattern (e.g., Down-regulated DEGs and SEX-specific), in favor of the former class. Among genes included in this latter category, we annotated two *AGAMOUS*-like genes (namely *AGL11, AGL62*) and several embryo defective proteins (Supplementary Table S7). The dataset represented by transcripts potentially involved in meiotic entry and sporogenesis included six down-regulated DEGs or SEX-specific variants and 5 genes displaying the opposite expression pattern (including Up-regulated DEGs and APO-specific).

**FIGURE 4:**
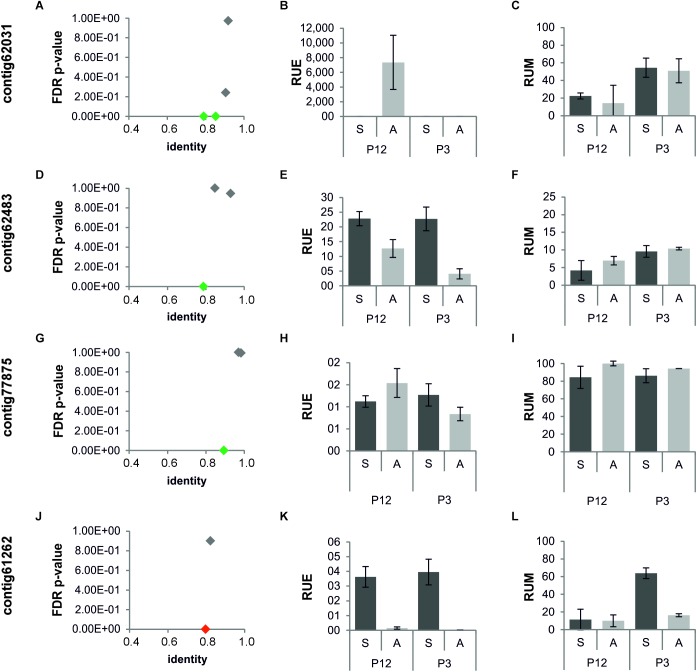
Sequence conservation, expression, and CpG methylation patterns of loci encoding for TEs and characterized by the expression of multiple transcript variants. **(A–C)** Contig62031; **(D–F)** contig62483; **(G–I)** contig 77875; **(J–L)** contig61262; **(A,D,G,J)** sequence identity and FDR *p*-value are plotted in the x and y axes, respectively. **(B,E,H,K)** Quantitative Real-Time PCR results for DEGs annotated as TEs, co-mapping into the same loci. **(C,F,I,L)** Methylation levels estimated for the same loci. Green diamonds: DEGs up-regulated or displaying APO-specific expression. Red diamonds: DEGs down-regulated or displaying SEX-specific expression. Gray diamonds: transcripts displaying no transcriptional changes. Dark gray bars: gene expression and DNA methylation levels in sexual samples; light gray bars: gene expression and DNA methylation levels in aposporous samples. P12: pistils at stage 1 and 2 (pre-meiosis and meiosis). P3 pistils at stage 3 (early gametogenesis). RUE: relative units of expression. RUM: relative units of methylation.

Taking into consideration the massive presence of transcripts annotated as RNA-dependent DNA biosynthetic process in our sequence datasets (Table 4 and Supplementary Table S6), we wondered whether the differential expression of transposable elements (TEs)-like sequences was somehow associated to aposporous phenotype, in pre-meiotic ovules. To address this question, we aligned all DEGs (Bonferroni *p*-value ≤ 0.05) to the genome sequence, and isolated the genomic loci providing the best alignments (Supplementary Table S8). Although several (n: 5) transposon related sequences were predicted in the genomic loci matching the HAPPY locus, we did not find transcript variants matching these loci. The alignment of the entire ovule transcriptome to the genomic loci targeted by differentially expressed TEs provided a total 42 transcript variants, including: 13 Up-regulated, 3 APO-specific and 3 down-regulated transcripts, together with 23 transcripts displaying no significant expression differences between sexual and aposporous samples (Supplementary Table S8). The annotation of these 12 genomic loci by BLASTX confirmed their TEs nature. Remarkably, for as many as 8 out 12 loci, the transcript with the lowest FDR *p*-value associated gene expression, displayed the highest nucleotide diversity with respect to the genome reference (Figure 4 and Supplementary Table S8). Pearson correlation coefficients (PCC) ≥ 0.59 between FDR *p*-value and sequence identity were estimated for most of these genomic loci (N: 7 out of 8, Supplementary Table S8). Moving from these data, we selected four loci with significant PCC correlations and investigated their expression pattern and CpG DNA methylation at pistil level (Figure 4). The expression pattern observed by RNAseq assay in ovules was confirmed even at the level of pistils in three cases out of four. As for the DNA methylation pattern, although some methylation changes could be observed among the pistil developmental stages, we did not find any obvious correlation with the assessed expression data (Figure 4).

### Apomictic Ovules Display a Differential and Heterochronic Expression of Transcripts Involved in Epigenetic Regulation of Gene Expression and RNA Splicing

Following the identification of multiple TEs differentially expressed in our datasets, we annotated the ovule transcriptome for the identification of gene products potentially involved in epigenetic regulation of gene expression (Pikaard and Mittelsten Scheid, 2014). The adopted terms were RNA silencing, chromatin modification, DNA modification and RNA directed DNA methylation. Multiple DEGs and phenotype-specific transcripts felt in these classifications (Supplementary Table S9). On average, 3 transcript variants per locus were identified (minimum: 1, maximum: 4).

**FIGURE 5:**
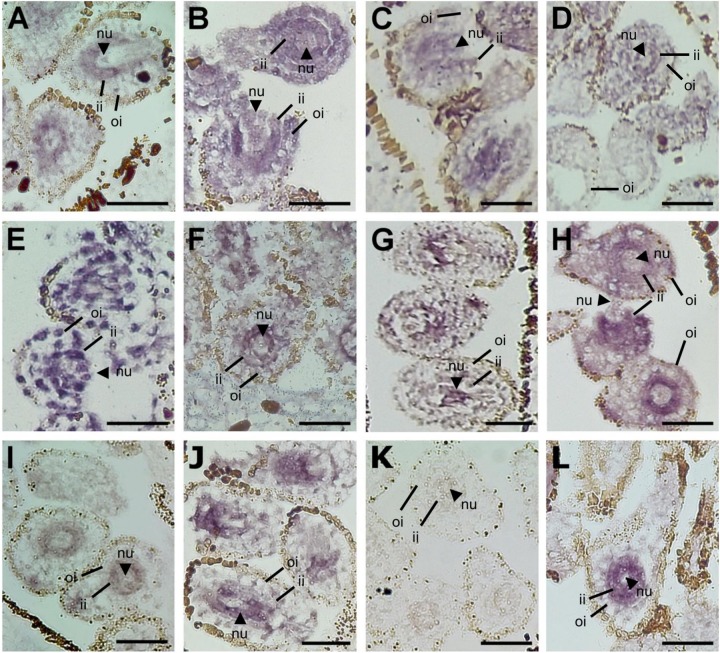
*In situ* RNA hybridization experiments in pistils collected from sexual and aposporous apomictic accessions. **(A,C,E,G,I,K)** Sexual genotypes; **(B,D,F,G,J,L)** aposporous apomict genotypes. **(A,B)** *HpIDN2*; **(C,D)** *HpFDM2*; **(E,F)** *HpFDM4*; **(G,H)** *HpRDR16*; **(I,J)** *HpLSD1*; **(K,L)** *HpTIL1*. Experiments were performed with the antisense probes, to hybridize mRNAs in sense orientation. The hybridization signal is indicated with the blue color. nu: nucellus; ii: internal integuments; oi: outer integuments. Scale bar: 50 mm.

The expression of six genes potentially involved in these epigenetic processes were tested by *in situ* hybridization assays (Figure 5). For all tested genes, hybridization signals were detected in the ovule nucellus, which is consistent with the sequencing of RNAs expressed in this cellular domain. However, hybridization signals were also detected in additional cellular domains of the ovule, including the integuments (Figure 5). In line with the RNAseq data, hybridization signals for *HpIDN2, HpFDM2, HpFDM4, HpRDM16* were detected in ovules collected from both phenotypes. Hybridization signals for the two genes *HpTIL1* and *HpLSD* were faint or undetectable in ovules collected from sexual plants. Furthermore, the hybridization patterns observed with the respective complementary probes suggest that transcription of selected genes occurs in both orientations (Supplementary Figure S2).

**FIGURE 6:**
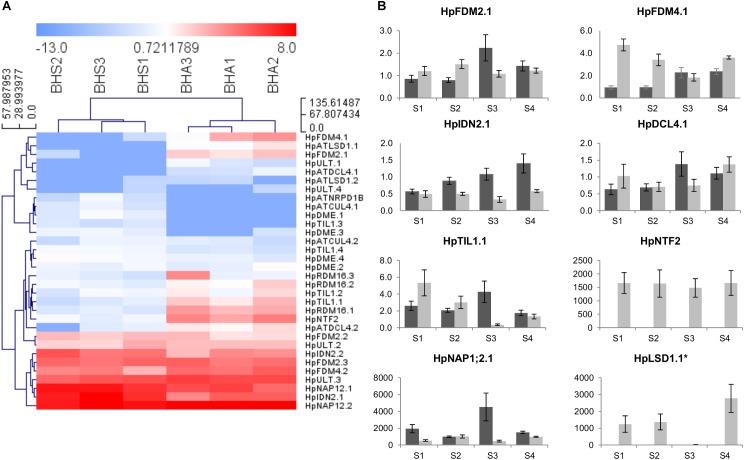
Expression of *H. perforatum* loci involved in epigenetic regulation of gene expression. **(A)** Heat map showing the expression of transcript variants in ovules dissected from sexual and aposporic accessions, as assessed by RNAseq. The HCL was performed by using Manhattan distances and average linkage clustering. **(B)** Quantitative Real-Time PCR results for a number of loci involved in epigenetic regulation of gene expression. Light gray bars: relative expression level in sexual pistils; dark gray bars: relative expression level in aposporous pistils. Pistil developmental stages: S1: pre-meiosis; S2: meiosis; S3–S4 early and late gametogenesis, respectively. Relative expression values are plotted on the vertical ax. Error bars indicate the standard error observed among the five biological replicates. Error bars indicate the standard error observed among the five biological replicates. ^∗^: the expression was detected in three apomictic samples out of five.

**FIGURE 7:**
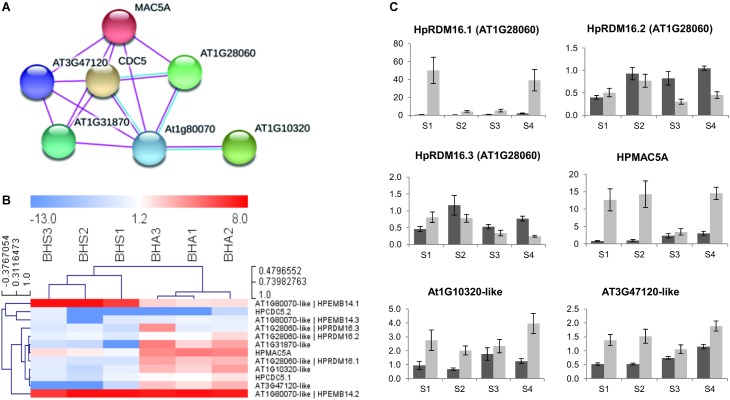
Expression of *H. perforatum* loci involved in RNA processing and splicing. **(A)** Network showing known interactions between proteins encoded by the same loci. Nodes represent *A. thaliana* orthologs, while edges represent protein–protein interactions. Only known interactions from curated databases (blue) or experimentally determined (pink) were considered. The average local clustering coefficient is 0.876. The PPI enrichment *p*-value is 5.8e-5. **(B)** Heat map showing the expression of transcript variants in ovules dissected from sexual and aposporic accessions, as assessed by RNAseq. The HCL was performed by using Manhattan distances and average linkage clustering. **(C)** Quantitative Real-Time PCR results and known interactions for a number of *H. perforatum* DEGs potentially involved in RNA processing. Light gray bars: relative expression level in sexual pistils; dark gray bars: relative expression level in aposporous pistils. Pistil developmental stages: S1: pre-meiosis; S2: meiosis; S3–S4 early and late gametogenesis, respectively. Relative expression values are plotted on the vertical ax. Error bars indicate the standard error observed among the five biological replicates.

As shown in Figure 6, the hierarchical clustering of samples according to the expression of annotated transcripts was consistent with the two phenotypes (Figure 6A). At the same time, the heat map underlined the extent to which expression variation was captured in our RNA-seq assembly and GE pipelines. qPCR analyses were performed to assess the expression of selected genes on broader range of developmental stages, ranging from pre-meiosis (stage S1) to the end of gametogenesis (stage S4). At stage S1, the pattern recorded by qPCR in pistils for *HpFDM4.1, HpNTF2, HpLSD1.1, HpTIL1.1*, and *HpNAP1;2.1* was in line with RNAseq data on LCM samples. Conversely, *HpFDM2.1, HpIDN2.1*, and *HpDCL4.1* displayed similar expression levels in S1 pistils collected from sexual and aposporous plants. Nevertheless, multiple transcripts involved in RNA silencing and/or RNA directed DNA methylation displayed heterochronic expression, with higher or comparable transcript levels in sexual pistils at stage 3 (early gametogenesis), and higher or comparable transcript levels in apomictic pistils at stages S1 and/or S2 (Figure 6B, 7C). This was true for *HpFDM2.1, HpFDM4.1, HpIDN2.1, HpTIL1.1*, and *HpDCL4.1* (Figure 6). Interestingly, the expression of *HpNAP1;2*, encoding for a nucleosome assembly protein involved in chromatin formation or chromatin remodeling, was modulated before and after meiosis (stages S1 and S3). According to the qPCR assays, the expression of *HpLSD1.1* and *HpNTF2*, encoding for the orthologs of a LYSINE-SPECIFIC HISTONE DEMETHYLASE and NUCLEAR TRANSPORT FACTOR 2, were nearly exclusively detected in aposporous pistils (Figure 6B). Our RNAseq investigations identified two differentially expressed transcripts displaying high sequence similarity with the Pre-mRNA-splicing factor 3-like *RDM16* and mapping on different genomic contigs (contig 62591 and contig 62634, respectively). We named these transcripts *HpRDM16.1* and *HpRDM16.3*. A third *RDM16*-like transcripts displaying no transcriptional changes in sexual and aposposporous ovules co-aligned with *HpRDM16.1* and was named *HpRDM16.2*. Accordingly, *HpRDM16.2* displayed no expression difference in S1 pistils of the two phenotypes whereas the average expression level of *HpRDM16.3* in pistils collected at stage S1 was higher aposporous plants (Figure 7C). Regarding the expression of *RDM16.1* in aposporous pistils (Figure 7C), this was characterized higher expression in terminal stages of aposporous pistils and by a pronounced expression difference between central developmental stages, those embracing meiosis and early gametogenesis, and the most marginal ones (e.g., S1 and S4). These findings, together with the differential expression of two *RDM16*-like genes and their possible role in DNA methylation (e.g., *RDM16* stands for Reduced DNA Methylation 16) prompted us to ask whether transcriptional de-regulation of this gene might have a broader effect in the ovule transcriptome. In the heterologous system represented by the sexually reproducing *A. thaliana*, the protein RDM16 (AT1G28060) physically interacts with several proteins involved in RNA processing and splicing. We therefore searched our datasets for potential interactors of HpRDM16, involved in these processes (Figure 7B). The network represented in Figure 7A shows the potential interactors of HpRDM16 having at least one differentially expressed transcript variants in our RNAseq dataset (Figure 7B). The network has an average local clustering coefficient of 0.876 and a protein–protein interaction (PPI) enrichment *p*-value of 5.8e-5, indicating that proteins included in this network are at least partially biologically connected, as a group. Beside the known protein–protein interactions, the network reports a significant enrichment (FDR *p*-value: 8.54e-06) in proteins involved in RNA splicing (KEGG map: ath03040). The clustering of corresponding *H. perforatum* transcripts based on ovule-RNAseq data is consistent with the conservation of their biological connection in this latter species (Figure 7B). In line with the RNAseq data, higher expression in aposporous pistils at stage S1 was recorded by qPCR for *HpMAC5A, AT1G10320*-like and *AT3G47120*-like. Intriguingly, genes included in this group seemed to be communized by marked differences in gene expression for terminal stages and very low expression differences (if some) between sexual and aposporous pistils at stage S3, corresponding to the onset of gametogenesis (Figure 7C). The identification of network of potentially interacting proteins involved in RNA binding and splicing and encoded by transcripts up-regulated in the aposporous ovule nucellus prompted us extend of qPCR assays on additional transcripts annotated with these terms (Supplementary Figure S3). qPCRs were performed to target the expression of a putative Serine/arginine-rich SC35-like splicing factor *HpSCL28*, together with *HpSRP54, HpPUM3* and the F-box family protein *HpSKIP22*-like. In line with the RNAseq data, higher expression in aposporous S1 pistils was detected for *HpSCL28* and *HPSRP54* and *HpSKIP22*-like. Differential expression for the *HpPUM3*-like transcript, which encodes for a PUMILIO homolog 3 involved in the regulation of mRNA stability by binding the 3′-UTR of target mRNAs, was only detected in pistils at stage S4. Altogether, validation of RNAseq profiles on pistils collected at a comparative developmental stage provided consistent amplification profiles for 70% of investigated transcripts (N. 16 out of 22).

It is worth noting that expression of the *A. thaliana* orthologs of these genes is detectable in different plant structures. Within flowers it is generally higher at early developmental stages (Flower stage 9), it is enriched in pistils (referred as carpels, flower stages 12 and 15), and among the different pistil areas, it is more abundant in the ovary, which is pistil part that bears the ovules^12^.

## Discussion

This study is aimed at investigating gene expression variation potentially associated to the aposporous developmental program by focusing on the pre-meiotic ovule nucellus, which is the cellular domain primarily involved into the differentiation of precursors of meiocytes and aposporous embryo sacs. Transcriptomic investigations were performed on a wide range of aposporous accessions, and by adopting a bulking strategy. This experimental design was adopted to focus on transcripts and meaningful biological processes conserved across different apomictic accessions. Hence, high coverage sequencing reactions (nearly 90M pair-end reads/library) and stringent criteria for the selection of phenotype-specific transcripts and DEGs were adopted to address the possible drawback represented by the adoption of multiple plant accessions deriving different geographical areas. Taken together, the size (N: 67,000) and N50 of the assembled transcriptome (N50: 814 bp) suggests that our *de novo* transcriptome assembly retains some redundancy. Nonetheless, our gene expression analysis identified nearly 400 DEGs (Bonferroni *p*-value ≤ 0.05) and up to 1832 transcripts characterized by phenotype-specific expression. The expression of six selected DEGs in the ovule nucellus was confirmed by *in situ* hybridization assays performed on both sexual and aposporous tissues. However, hybridization signals for the assayed genes were also detected in other ovule domains (e.g., internal integuments), indicating that expression of the selected DEGs is not restricted to the nucellus.

To better exploit our transcriptome data and investigate the extent to which single loci were represented by multiple variants, we sequenced the genome of an *H. perforatum* diploid sexual accession. In its current version, the draft sequence of the *H. perforatum* genome is about 350 Mb in size, with a scaffold N50 of about 63 kb, indicating that half of the genome sequence is in contigs larger than 63 kb. A preliminary investigation concerning the completeness of the assembly estimated an 85.7% completeness, 12.8% of duplicated and about 5% of fragmented benchmarking universal single-copy orthologs (BUSCOs). About 9% of investigated universal single-copy orthologs were missing from our assembly. The *in silico* mapping of genes located in the HAPPY locus associated to apospory in *H. perforatum* identified of nine contigs sharing high sequence similarity (98% within gene regions) with the corresponding BAC clone (HM061166). The largest assembled contig (62786) corresponded to a large sequence portion of HM061166 in between the two genes *PAT1* and *RINGH2* (Supplementary Table S5). As expected, synteny and collinearity were perfectly conserved between gene loci annotated in contig 62786 and the corresponding sequence portion of HM061166. Noteworthy, the *in silico* mapping of corresponding gene regions permitted the annotation of a 50 kb sequence portion upstream the gene locus HK1 included in contig 62819, which likely represents a portion of the genome sequence upstream of the HAPPY locus in the sexual *H. perforatum* genome. The alignment of transcript variants to the gene loci annotated in the HAPPY locus identified 24 genes expressed in pre-meiotic ovule nucellus. *ARIADNE7* was among the predicted genes with the highest expression in sexual and aposporous datasets. However, no transcriptional difference was detected for this gene and the only DEG annotated in this locus encoded for a light harvesting chlorophyll a/b-binding protein (Lhcb2), located about 40 kb upstream of *HK1* (Supplementary Table S5).

The sequencing and assembly of a reference genome permitted the efficient annotation of identified DEGs and the identification of co-expressed transcript variants, defined as transcript sequences co-aligning to the same genomic sequence (Figure 3). Based on these investigations, we detected multiple variants with alternative expression patterns for about one third of identified DEGs and phenotype-specific transcripts, with an average number of about two transcripts per locus (DEGs: 1.8; phenotype-specific: 1.9). However, we are aware that computational assumptions adopted for their selection might have led to an underestimation on the number of transcript variants. Detailed investigations on a subset of gene loci encoding for TEs revealed that gene loci with the highest nucleotide diversity with respect to the genome reference were more likely to be differentially expressed (N: 8 loci out of 12), as indicated by the lower FDR *p*-value associated gene expression variation. Although no additional investigations were performed to address the extent of nucleotide diversity among transcript variants, the co-expression of multiple transcripts with alternative expression pattern might suggests high plasticity in mRNA transcription and processing.

Our comparative GE analysis underlined a clear disproportion between transcripts displaying higher expression in aposporous-derived ovule nucellus (including Up-regulated DEGs and APO-specific) and transcripts with the opposite expression pattern (e.g., Down-regulated DEGs and SEX-specific). Taken together, and without a clear correlation with technical or methodological features, it is likely that a correlation exists between the aposporous developmental pathway in *H. perforatum* and the observed enhanced expression in ovules of aposporous accessions. The observed disproportion between transcripts displaying higher expression in aposporous-derived ovules and transcripts with the opposite expression pattern, is consistent with the hypothesis that aposporous plants are subjected to alternative transcriptional (mediated by trans-acting factors) or post-transcriptional regulation of gene expression in the ovule nucellus.

By focusing on single biological pathways, the transcriptome of pre-meiotic aposporous nucellus was enriched in GO terms related to RNA-dependent DNA biosynthetic process (GO:0006278), while terms related to the RNA processing (GO:0006396) and epigenetic regulation of gene expression (GO:0040029) were significantly depleted. Accordingly, our GE study shows massive differences in the expression of several genes encoding for TEs. Enrichment of TEs -related GO-terms were identified in all comparisons, with the only exception of transcripts exclusively expressed in sexually-derived samples (Supplementary Table S6). The possible association between sequence conservation and differential expression observed for a number of TEs might lead to speculate that their correspondent loci are located within the *Hypericum* apomixis controlling region, which is currently represented by the HAPPY locus (Schallau et al., 2010). However, the differentially expressed TEs did not map into the HAPPY locus and no expression was recorded for the TEs annotated within the HAPPY locus. At the same time, the distribution of transcriptionally modulated TEs across the genome appears to be widespread. As an example, the genomic area represented by considering only TEs whose expression is enriched in aposporous samples (Bonferroni *p*-value ≤ 0.05) accounts for 12 contigs, with an overall size of about 3.5 Mb (nearly 1% of the assembled genome). Taken together, these data suggest that massive differential expression of TEs-related genes might be associated to the mis regulation of cellular processes involved in post-transcriptional regulation of TEs.

As apomixis could be related to heterochrony in the expression of reproductive processes (Grimanelli et al., 2003), we annotated all DEGs according to their possible involvement in reproductive processes. Only a few DEGs potentially associated with sporogenesis and gametogenesis were identified (Supplementary Table S7). The low number of DEGs potentially associated with reproduction is in line with the pre-meiotic developmental stage adopted for the laser capture microdissections. More in details, samples collected from aposporous accessions were characterized by higher transcript levels for the orthologs of the Arabidopsis gene BEL-like homeodomain 1 (*BLH1*), which is involved in cell specification. Transcriptional studies on the aposporous model plant *Hieracium prealtum* have shown that a putative homolog of the *BEL*-like *EOSTRE* is expressed in microdissected aposporous initials and early aposporous embryo sacs (Okada et al., 2013). Interestingly, embryo sac development in aposporous *H. perforatum* (Galla et al., 2011) and *Hieracium* spp. (Okada et al., 2013) accessions shows defects in nuclear migration and cellularization that might resemble those associated to the ectopic expression of BLH1 in *A. thaliana* embryo sac (Pagnussat et al., 2007). Furthermore, multiple transcript variants with antagonistic expression patterns were associated to the *HpTIL1* gene, encoding for the catalytic subunit of DNA pol ε, whose mutation in Arabidopsis display pleiotropic phenotypes including reduced number of ovules, abnormally developing ovules, and reduced fertility (Jenik et al., 2005). *ATMYB66*, another transcription factor displaying higher expression in aposporous samples, is involved in epidermal cell fate specification in *A. thaliana*, although this phenotype has been documented in roots and hypocotyls (Cheng et al., 2014). Interestingly, results have shown that MYB66 physically interacts with several splicing factors including the protein encoded by AT2G32600, the pre-mRNA-splicing factor ISY1 (AT3G18790) and an ATP-dependent RNA helicase encoded by AT3G26560. Among these interactors, only the orthologous ISY1 (AT3G18790) was up-regulated in aposporous samples (Supplementary Table S4; FDR *p*-value: 3.73e-3). However, several DEGs with the potential to interact at protein level and likely involved in RNA splicing were detected in our datasets (Figure 7 and Supplementary Table S10). These include the orthologs of AT1G10320, AT3G47120, BUD13 (AT1G31870) CDC5 (AT1G09770), EMB14 (AT1G80070), MAC5A (AT1G07360), and RDM16 (AT1G28060). The PPI network represented by the gene products of these DEGs has a protein–protein interaction enrichment *p*-value of 5.8e-5, indicating that proteins included in this network are at least partially biologically connected, as a group. Consistent with the gene annotations, the network has a significant enrichment (FDR *p*-value: 8.54e-06) in proteins involved in RNA splicing (KEGG map: ath03040). Pre-mRNA splicing is an essential process required for the expression of most eukaryotic genes. Alternative splicing produces multiple mRNAs from the same gene through variable selection of splice sites during pre-mRNA splicing. Noteworthy, more than 60% of Arabidopsis intron containing genes displays alternative slicing (Marquez et al., 2012) and the percentage of alternatively spliced genes in humans is about 95% (Pan et al., 2008). Splicing is carried out by a macromolecular machinery termed the spliceosome, which senses the splicing signals and catalyzes the removal of introns from pre-mRNAs. Results in Arabidopsis and corn have shown that alternative RNA splicing is needed for cell differentiation, development, and plant viability (Fouquet et al., 2011). Moreover, the knockout of *MAC5A* in Arabidopsis displays severe developmental defects including dwarfism, delayed growth, abnormal floral organs, and sterility (Monaghan et al., 2010). Regarding the expression of DEGs involved in RNA splicing before the onset of meiosis, the higher average gene expression detected in aposporous pistils by qPCR was in line with the higher expression detected with the RNAseq analysis on LCM samples. This could indicate that expression is enriched in the ovule nucellus or that regulation mechanisms for these genes are shared throughout the pistil. Furthermore, according to our time-course qPCR assays, the expression variation observed among aposporous and sexual pistils was remarkably high for terminal developmental stages and much lower in correspondence of the onset of gametogenesis (stage S3). Noteworthy, an enrichment in expression of cell cycle related genes was also documented in *Hieracium prealtum* AI cells and EAE sacs (Okada et al., 2013) and *Boechera gunnisoniana* apomictic germlines (Schmidt et al., 2014) and an ortholog of AT1G45231 (*TGS1*), encoding a trimethyl guanosine synthase which has a dual role in splicing and transcription, is increasingly overexpressed in sexual plants from pre-meiosis to anthesis in *Paspalum notatum* (Siena et al., 2014).

In addition to pre-mRNA splicing, splicing factors might also play important roles in other biological processes, including sRNA production (Zhang et al., 2013). Hence, the Arabidopsis ortholog of HpCDC5, which was up-regulated in our aposporous samples (Figure 7), positively regulates post-transcriptional processing and/or transcription of primary microRNA transcripts (Lin et al., 2007; Zhang et al., 2013). Furthermore, screenings for the identification of genes involved in RNA-directed DNA methylation (Ausin et al., 2012; Dou et al., 2013; Huang et al., 2013; Du et al., 2015) demonstrated that several pre-mRNA splicing factors, including RDM16, act at different steps in the RdDM pathway. In our study, two orthologs of the Arabidopsis pre-mRNA splicing factor RDM16 were associated to transcript variants significantly up regulated in aposporous micro dissected samples and pistils at different developmental timepoints. Results have shown that RDM16 regulates the overall methylation of TEs and gene-surrounding regions, and preferentially targets Pol IV-dependent DNA methylation loci and the ROS1 target loci (Huang et al., 2013). Arabidopsis *rdm16* mutants are affected in several aspects of plant development, including the viability of both female and male gametes (Huang et al., 2013). Interestingly, the small nuclear ribonucleoprotein Prp4p-related (LACHESIS), which is involved in a mechanism that prevents accessory cells from adopting gametic cell fate within the female embryo sac, is among the known interactors of the Arabidopsis RDM16^13^. *HpRDM16.1* and *HpRDM16.3* were not the only upregulated transcripts involved in epigenetic regulation of gene expressions by RdDM. Differential expression was also detected for genes operating in RNA silencing, RNA-directed DNA methylation (*HpDCL4.1, HpIDN2.1, HpFDM2.1, HpFDM4.1, HpNTF2.1*) and histone and chromatin modification processes (*HpLSD.1* and *HpNAP1;2.1*). These transcriptional changes are consistent with a role of these processes in regulating cell fate determination in the ovule, as indicated by genetic studies in *A. thaliana* and maize (Garcia-Aguilar et al., 2010; Olmedo-Monfil et al., 2010; Armenta-Medina et al., 2011; Singh et al., 2011). qPCR reactions performed on genes potentially associated RNA silencing and RdDM only partially validated the RNAseq expression data. Nevertheless, as the down-regulation of *HpIDN2* in aposporous *H. perforatum* pistils was also detected on a previous study (Galla et al., 2017), we hypothesize that validation of RNAseq data focused on ovule nucellus in pistils might be affected by the amplifications of multiple variants with contrasting expression pattern (Supplementary Table S9) and/or transcriptional noise associated to expression of these genes in other pistils cellular domains. Consistently, microarray data in Arabidopsis have shown that expression for these genes is higher in pistils (referred as carpels, flower stages 12 and 15) and enriched but restricted to the ovary, which is pistil part that bears the ovules. Noteworthy, the putative homologs of Arabidopsis genes involved in chromatin function and gene silencing via small RNA pathways, including a *DICER-2* like gene, were enriched in *Hieracium prealtum* aposporous initials (Okada et al., 2013). Our time-course GE analysis on pistils collected at multiple developmental timepoint ranging from pre-meiosis to late gametogenesis revealed heterochronic expression for several genes involved in RNA splicing, RNA silencing and RdDM, includin *HpFDM2, HpFDM4* and *HpTIL1, HpMAC5A, RDM16.2* and the orthologs of *AT1G10320* and *AT3G47120*, among others. For these genes, the higher or comparable expression in sexual pistils in correspondence to the onset of gametogenesis was accompanied by an inverted expression pattern in preceding developmental stages (Figure 5, 6 and Supplementary Figure S3). Even though the heterochronic expression pattern is currently documented for a very narrow set of genes in this species, the occurrence of heterochronic expression of genes involved in reproductive processes in apomictic species has been documented in several model species (Grimanelli et al., 2003; Sharbel et al., 2010). Remarkably, a an ortholog of the Arabidopsis NUCLEAR TRANSPORT FACTOR 2 (NTF2) family protein which is up-regulated in the aposporous nucellus (Bonferroni *p*-value: 9.5E-09) displayed constitutive expression in aposporous pistils and nearly no amplification signals in sexual pistils. *NTF2* encodes for an RNA binding (RRM-RBD-RNP motifs) protein predicted to bind ssRNA (Allain et al., 2000) and involved in RdDM (Parida et al., 2017). Results have shown that the Arabidopsis NTF2 interacts with the methyl CpG binding domain MBD6, involved RNA-mediated gene silencing (Parida et al., 2017). Other known interactors of ATMBD6 are the two proteins: AGO4, which is a member of a class of PAZ/PIWI domain containing proteins involved in siRNA mediated gene silencing, and the histone deacetylase AtHDA6, which is another essential component of in RNA-directed DNA methylation involved in the silencing of TEs by modulating DNA methylation and histone acetylation (Liu et al., 2009). The expression of genes encoding for ARGONAUTE proteins did not vary significantly in our datasets. However, we did find significant differences in the expression of multiple genes encoding for AGO4 interacting proteins. Among these, significant transcriptional differences were detected for transcripts encoding for the orthologs of the XH/XS domain containing proteins: IDN2, FDM2, and FDM4. The interaction of IDN2 with FDM-like proteins is thought to be required for RdDM (Ausin et al., 2009; Zheng et al., 2010). In our current understanding on their molecular function, IND2, FDM2 and possibly FDM4, together with AGO4, are required for the association of DOMAIN REARRANGED METHYLTRASFERASE 2 (DRM2) with lncRNAs, at RdDM targeted loci (Bohmdorfer et al., 2014). Furthermore, at chromatin level, IDN2 physically interacts with a core subunit of the SWI/SNF complex (SWI3B) and mediate transcriptional silencing by guiding the SWI/SNF complex and establishing positioned nucleosomes on specific genomic loci (Zhu et al., 2013). Interestingly, the knockout of the two Arabidopsis LYSINE-SPECIFIC HISTONE DEMETHYLASE: LSD1 and LSD2 affects CHH methylation levels at a subset RdDM target loci (Greenberg et al., 2013). However, any functional correlation existing between IDN2 and the transcriptionally modulated LSD1 and NUCLEOSOME ASSEMBLY PROTEIN 1;2 (NAP1;2) at chromatin level is currently not clear and deserves additional investigations. Noteworthy, an enrichment of functions related to epigenetic regulatory pathways, including histone H3K4 demethylation and maintenance of DNA methylation were also reported aposporous initials of apomictic *Boechera gunnisoniana* (Schmidt et al., 2014), suggesting that epigenetic regulation of gene expression is a common feature of the aposporous reproductive strategy in different species.

## Conclusion

Pre-meiotic ovule nucellus of aposporous plants are characterized by variations in the expression of a great number of genes, including TEs-related loci, which appears to be widespread across the genome. About one third of gene loci associated to DEGs and phenotype-specific transcripts were characterized by the co-expression of multiple transcript variants with antagonistic expression patterns. Our comparative gene expression analysis detected a clear disproportion between transcripts displaying higher expression in the nucellus of aposporous plants and transcripts with the opposite expression pattern. This observation is consistent with the hypothesis that aposporous nucellar cells are subjected to alternative transcriptional (mediated by trans-acting factors) or post-transcriptional regulation of gene expression in the ovule nucellus. Accordingly, DEGs were enriched or depleted in ontological terms related to RNA-dependent DNA biosynthetic process, RNA processing and epigenetic regulation of gene expression, including gene silencing by RNA and ncRNA metabolic process. The differential expression of multiple TEs-related sequences in ovules of aposporous accessions is in line with a functional association between apospory and the RNA directed DNA methylation pathways. Several genes encoding for potentially interacting proteins involved in pre-mRNA splicing were differentially expressed in the ovule nucellus, as well as terminal stages of pistil development. In addition to pre-mRNA splicing, DEGs involved in this process might also play important roles in sRNA production and RNA-directed DNA methylation. Noteworthy, several genes involved RNA-directed DNA methylation were also found differentially expressed in pre-meiotic aposporous nucellus and pistils collected at multiple developmental timepoints. These expression differences, together with GO enrichment analysis and the massive differential expression of TEs in pre-meiotic ovules are consistent with a deregulation of small RNA mediated DNA methylation in the ovule nucellus of aposporous *H. perforatum*.

## Author Contributions

GB, FP, and GG conceived the study. GG carried out the computational analysis and drafted the manuscript. AB performed all validations. MB, SG, and FP performed the LCM and prepared the RNA for the sequencing reactions. All authors helped to draft the manuscript and read and approved the final manuscript.

## Conflict of Interest Statement

The authors declare that the research was conducted in the absence of any commercial or financial relationships that could be construed as a potential conflict of interest.

## Footnotes

1 www.ortobotanicopd.it/en

2 http://www.ncbi.nlm.nih.gov/

3 https://github.com/vsbuffalo/scythe

4 https://github.com/najoshi/sickle

5 http://www.uniprot.org/

6 http://www.geneontology.org/

7 http://www.BLAST2go.org

8 goslim_plant.obo

9 http://amigo.geneontology.org/amigo

10 http://mev.tm4.org/

11 https://www.ncbi.nlm.nih.gov/geo/query/acc.cgi?acc=GSE128923

12 http://bar.utoronto.ca/ (Supplementary Table S11)

13 https://string-db.org/cgi/network.pl?taskId=CSYEOSdK3rb7

## Supplementary Material

FIGURE S1 Ontological annotation of the *H. perforatum* ovule transcriptome. Blue bars: biological processes (level 2); Green bars: molecular functions (level 3). For each annotation, the number of sequences is indicated in the horizontal ax and expressed as 1000 sequences. Annotations were clustered by using the plants-generic GO-slim function.

FIGURE S2 *In situ* RNA hybridization experiments in pistils collected from apomictic and sexual genotypes. **(A,C,E,G,I,K)** Sexual genotypes; **(B,D,F,G,J,L)** aposporous apomictic genotyes. **(A,B)** *HpIDN2*; **(C,D)** *HpFDM2*; **(E,F)** *HpFDM4*; **(G,H)** *HpRDR16*; **(I,J)** *HpLSD1*; **(K,L)** *HpTIL1*. Experiments were performed with the sense probes, to hybridize mRNAs in antisense orientation. Hybridization signals are indicated with the blue color. nu: nucellus; ii: internal integuments; oi: outer integuments. Scale bar: 50 μm.

FIGURE S3 Expression of *H. perforatum* transcripts involved in RNA binding and protein ubiquitination. Bars represent quantitative Real-Time PCR results expressed as relative units of expression. Light gray bars: relative expression level in sexual pistils; dark gray bars: relative expression level in aposporous pistils. Pistil developmental stages: S1: pre-meiosis; S2: meiosis; S3–S4 early and late gametogenesis, respectively. Relative expression values are plotted on the vertical ax. Error bars indicate the standard error observed among the five biological replicates.

TABLE S1 Origins of *H. perforatum* samples. For each plant accession, the description, the genealogy, the origin, the ploidy and the degree of apomixis is indicated. The RNA sample ID is reported only for plant accessions adopted for the RNAseq analysis. Apospory expressed as a percentage was determined by flow cytometric screening of 48 single seeds. For details on the origin and composition of experimental populations, please refer to Schallau et al. (2010).

TABLE S2 Summary of mRNA-seq data. For each library, the total number of reads as well as the number of mapped and unmapped reads are reported.

TABLE S3 Transcripts displaying differential expression in *H. perforatum* ovules (Bonferroni *p*-value correction). For each locus ID, transcript variants displaying no significant expression differences between sexual and aposporous samples are also reported. For each transcript, the locus ID in our reference genome, the alignment start/end and the number of allele variants are also reported. repBD: annotations regarding reproductive processes (Galla et al., 2017). GOrep: ontological annotations related to reproductive processes. Gene annotations were downloaded from AmiGO2 (http://amigo.geneontology.org/amigo). Epigenetic mod: annotations related to genes involved in epidenetic regulation of gene expression as reported by Pikaard and Mittelsten Scheid (2014). The average expression of each transcript in sexual (sexual – means) and aposporous (aposporous – means), together with Bonferroni and FDM *p*-values corrections associated to GE analyses are also reported.

TABLE S4 Transcripts annotated as phenotype-specific. Phenotype-specific transcripts were defined as transcripts expressed in all samples of a given phenotype and absent in all samples of the opposite phenotype. For each locus ID, transcript variants displaying no significant expression differences between sexual and aposporous sample as are also reported. For each transcript, the locus ID in our reference genome, the alignment start/end and the number of allele variants are also reported. repBD: annotations regarding reproductive processes (Galla et al., 2017). GOrep: ontological annotations related to reproductive processes. Gene annotations were downloaded from AmiGO2 (http://amigo.geneontology.org/amigo). Epigenetic mod: annotations related to genes involved in epigenetic regulation of gene expression as reported by Pikaard and Mittelsten Scheid (2014). The average expression of each transcript in sexual (sexual – means) and aposporous (aposporous – means), together with Bonferroni and FDM *p*-values corrections associated to GE analyses are also reported.

TABLE S5 Annotation and expression of genes located into the HAPPY (Hypericum APOSPORY) locus. For each contig ID and annotated locus, the table reports on the gene name, gene coordinates (locus start|end) and the ID of associated transcript variants. Genes annotated in the BAC clone HM061166 are indicated by using the bac ID followed by the gene name and percentage sequence identity with the aligned portion of the corresponding contig. Percent sequence identity is reported in bracket. For each transcript ID, the length of the alignment, the identity in the aligned portion of the sequence are indicated. The average expression of each transcript in sexual (sexual – means) and aposporous (aposporous – means), together with Bonferroni and FDM *p*-values corrections associated to GE analyses are also reported.

TABLE S6 GO Enrichment analysis. For each cluster of DEGs the over/under represented GO term (GO-ID), the ontological vocabulary (Ontology), the description of the GO term, the FDR corrected *p*-value and the *p*-value are reported. The number of annotated and non-annotated sequences in each test and reference datasets is also indicated.

TABLE S7 Annotation and expression of reproductive-related DEGs in ovules collected from sexual and aposporous accessions. For each locus ID, transcript variants displaying no significant expression differences between sexual and aposporous samples are also reported. For each transcript, the locus ID in our reference genome, the alignment start/end and the number of allele variants are also reported. repBD: annotations regarding reproductive processes (Galla et al., 2017). GOrep: ontological annotations related to reproductive processes. Gene annotations were downloaded from AmiGO2 (http://amigo.geneontology. org/amigo). Epigenetic mod: annotations related to genes involved in epidenetic regulation of gene expression as reported by Pikaard and Mittelsten Scheid (2014). The average expression of each transcript in sexual (sexual – means) and aposporous (aposporous – means), together with Bonferroni and FDM *p*-values corrections associated to GE analyses are also reported.

TABLE S8 Nucleotide diversity and expression pattern of TEs expressed in *H. perforatum* ovules. For each locus ID, transcript variants displaying no significant expression differences between sexual and aposporous samples are also reported. For each transcript, the locus ID in our reference genome, the alignment start/end and the number of allele variants are also reported. aln pident: percentage sequence identity in the aligned portion of the transcript. PCC: Pearson correlation coefficients between aln_pident and FDR *p*-value.

TABLE S9 Expression pattern of transcripts potentially involved in epigenetic regulation of gene expression. For each locus ID, transcript variants displaying no significant expression differences between sexual and aposporous samples are also reported. For each transcript, the locus ID in our reference genome, the alignment start/end and the number of allele variants are also reported. repBD: annotations regarding reproductive processes (Galla et al., 2017). GOrep: ontological annotations related to reproductive processes. Gene annotations were downloaded from AmiGO2 (http://amigo.geneontology.org/amigo). Epigenetic mod: annotations related to genes involved in epidenetic regulation of gene expression as reported by Pikaard and Mittelsten Scheid (2014). The average expression of each transcript in sexual (sexual – means) and aposporous (aposporous – means), together with Bonferroni and FDM *p*-values corrections associated to GE analyses are also reported.

TABLE S10 Expression pattern of transcripts annotated as RNA binding. For each locus ID, transcript variants displaying no significant expression differences between sexual and aposporous samples are also reported. For each transcript, the locus ID in our reference genome, the alignment start/end and the number of allele variants are also reported. repBD: annotations regarding reproductive processes (Galla et al., 2017). GOrep: ontological annotations related to reproductive processes. Gene annotations were downloaded from AmiGO2 (http://amigo.geneontology.org/amigo). Epigenetic mod: annotations related to genes involved in epidenetic regulation of gene expression as reported by Pikaard and Mittelsten Scheid (2014). The average expression of each transcript in sexual (sexual – means) and aposporous (aposporous – means), together with Bonferroni and FDM *p*-values corrections associated to GE analyses are also reported.

TABLE S11 Graphical Representation of *A. thaliana* log transformed clustered data. For each gene/probe ID, the table reports on the unique experiment ID (UNIQID), the research area, the plant growth stage, the tissue and Arabidopsis ecotype adopted for the expression study. For each gene, references to the manuscript table and/or figure are also reported. *A. thaliana* gene expression data were retrieved from the BAR Expression Browser (http://bar.utoronto.ca/affydb/cgi-bin/affy_db_exprss_browser_in.cgi) (Toufighi et al., 2005). The output displays the average expression of replicate treatments relative to average of the appropriate control. Expression data were downloaded as graphical representation of log transformed clustered data.

TABLE S12 Primers used in this study. For each target gene, the forward and reverse primers are reported. The purpose of each primer combination is also reported.
