# Supplementary figures and images for: Ovule Gene Expression Analysis in Sexual and Aposporous Apomictic Hypericum perforatum L. (Hypericaceae) Accessions
Source: Front Plant Sci. 2019 May 24;10:654. doi: 10.3389/fpls.2019.00654 (PMC6543059; doi:10.3389/fpls.2019.00654)

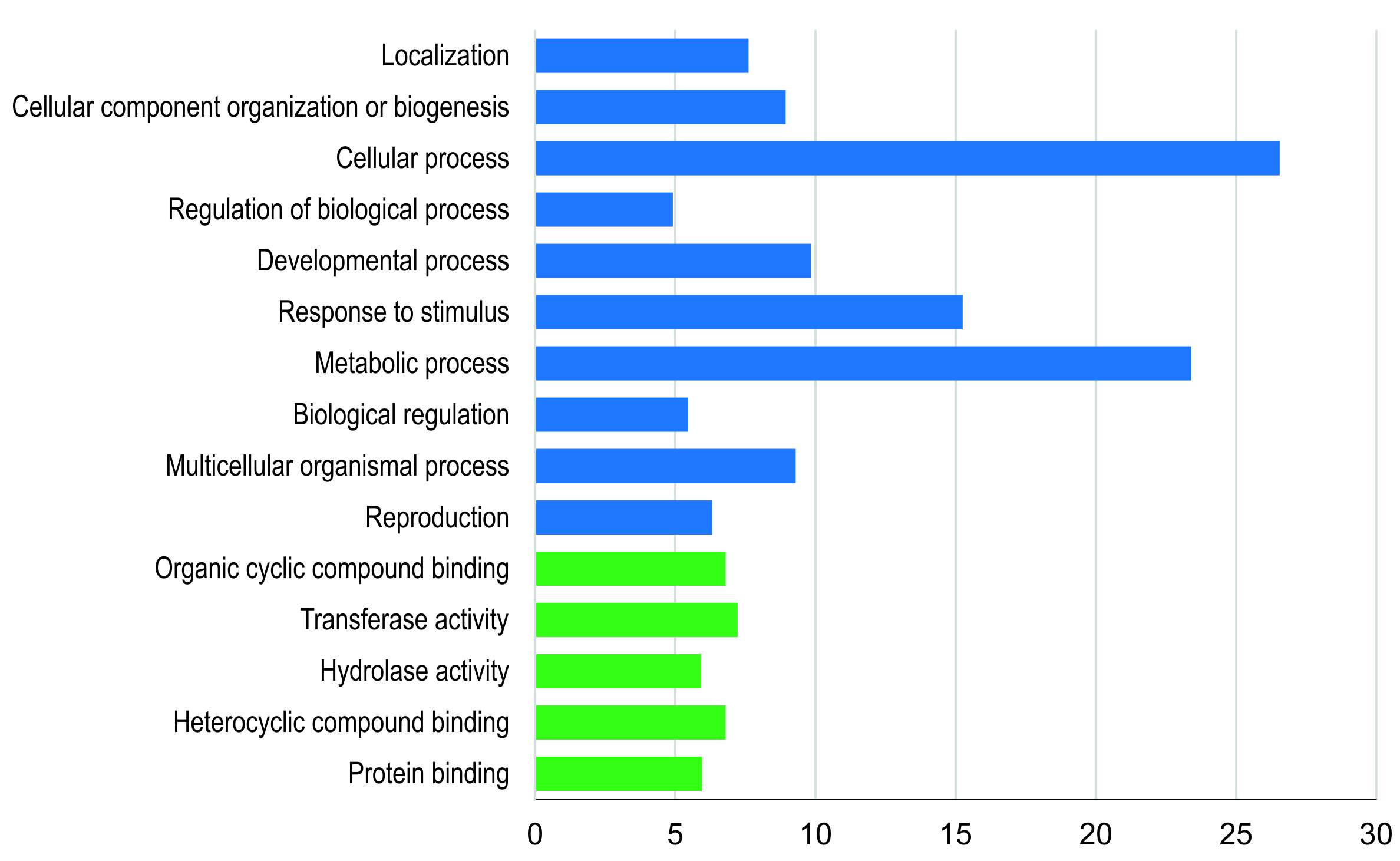

Supplement: FIGURE S1 — Ontological annotation of the H. perforatum ovule transcriptome. Blue bars: biological processes (level 2); Green bars: molecular functions (level 3). For each annotation, the number of sequences is indicated in the horizontal ax and expressed as 1000 sequences. Annotations were clustered by using the plants-generic GO-slim function. [file Image_1.TIF]

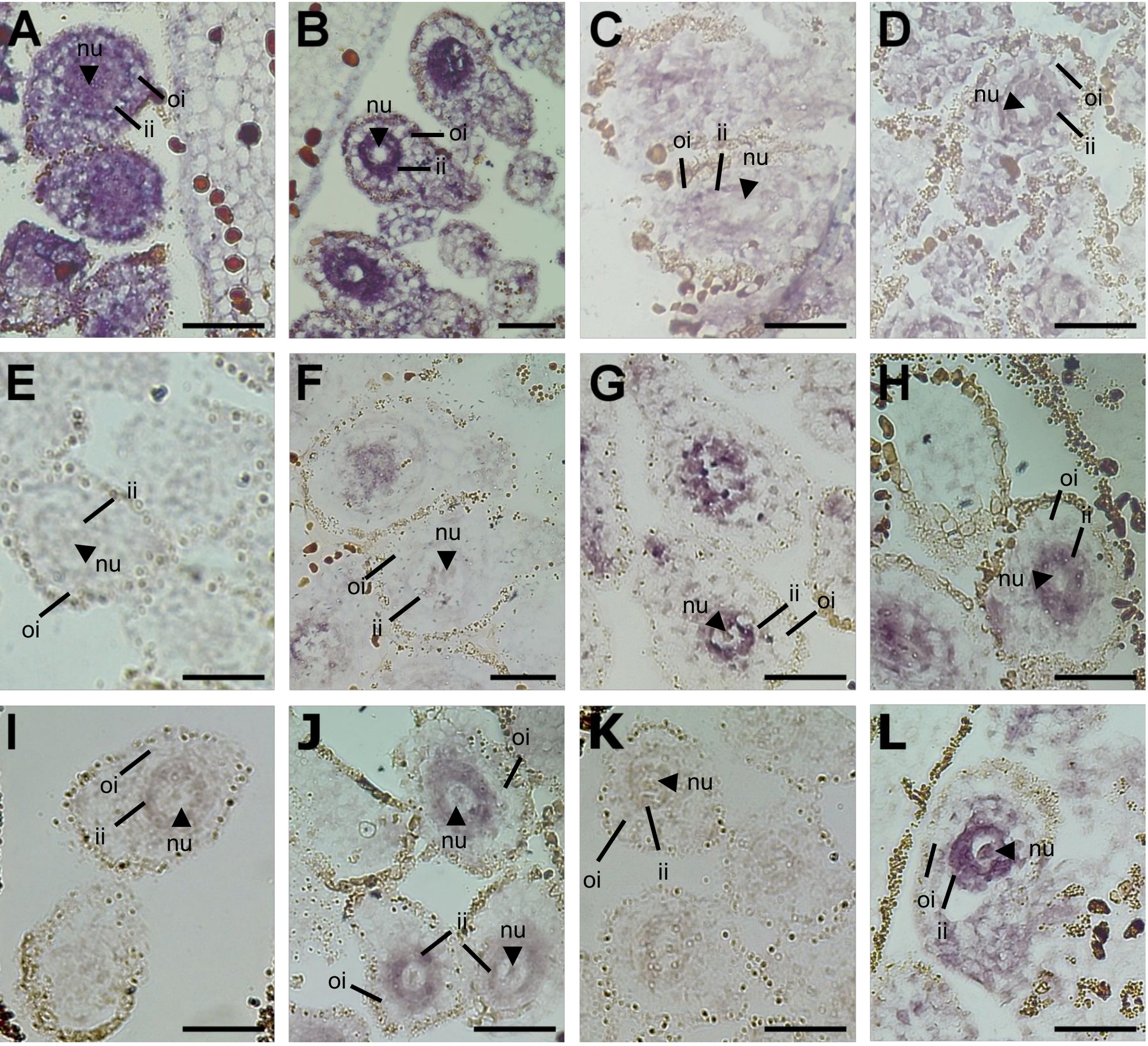

Supplement: FIGURE S2 — In situ RNA hybridization experiments in pistils collected from apomictic and sexual genotypes. (A,C,E,G,I,K) Sexual genotypes; (B,D,F,G,J,L) aposporous apomictic genotyes. (A,B) HpIDN2; (C,D) HpFDM2; (E,F) HpFDM4; (G,H) HpRDR16; (I,J) HpLSD1; (K,L) HpTIL1. Experiments were performed with the sense probes, to hybridize mRNAs in antisense orientation. Hybridization signals are indicated with the blue color. nu: nucellus; ii: internal integuments; oi: outer integuments. Scale bar: 50 μm. [file Image_2.TIFF]

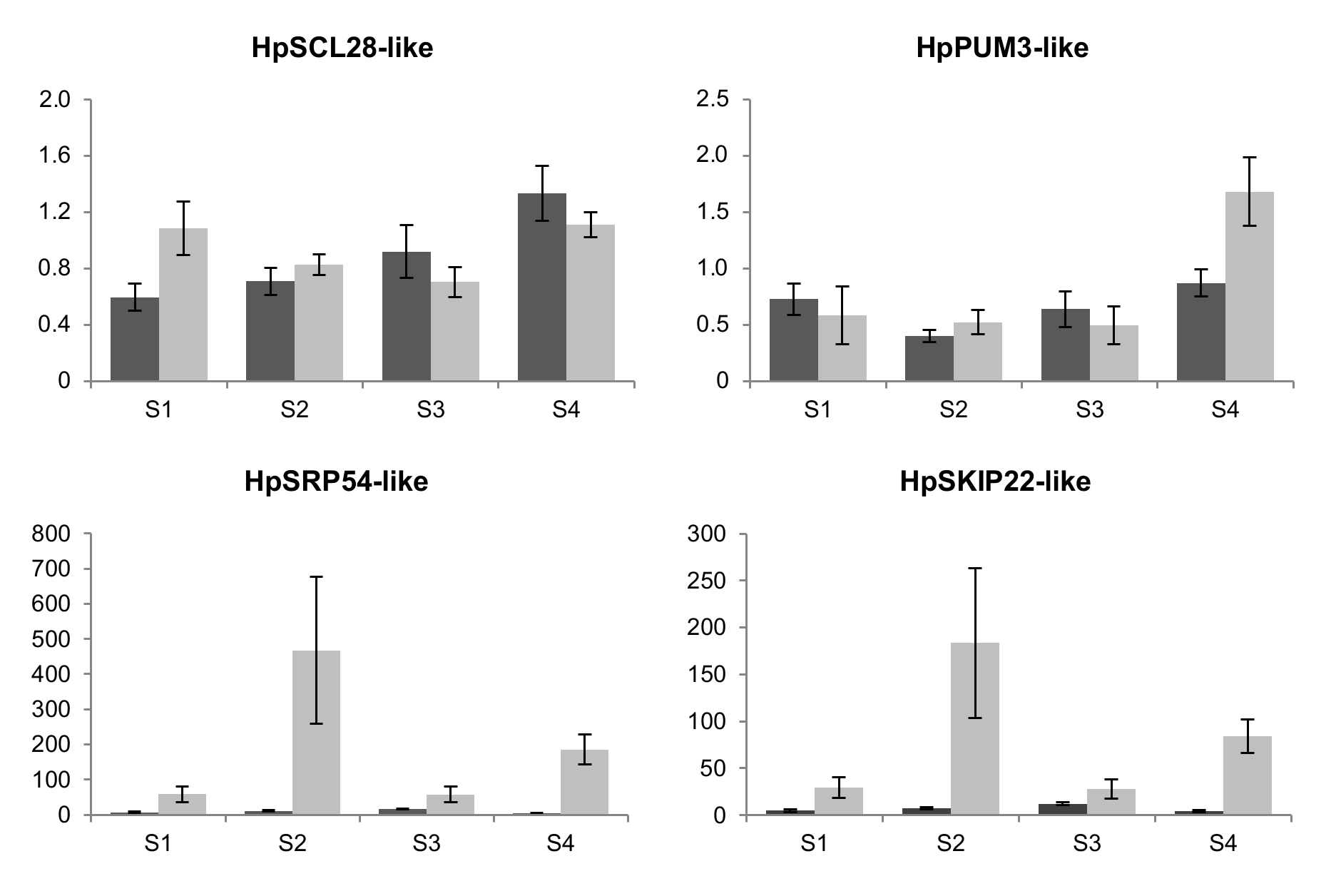

Supplement: FIGURE S3 — Expression of H. perforatum transcripts involved in RNA binding and protein ubiquitination. Bars represent quantitative Real-Time PCR results expressed as relative units of expression. Light gray bars: relative expression level in sexual pistils; dark gray bars: relative expression level in aposporous pistils. Pistil developmental stages: S1: pre-meiosis; S2: meiosis; S3–S4 early and late gametogenesis, respectively. Relative expression values are plotted on the vertical ax. Error bars indicate the standard error observed among the five biological replicates. [file Image_3.TIFF]
